# Vaccination, infection, and hybrid immunity: determinants of SARS-CoV-2 IgG antibody levels and protection in Quzhou, China

**DOI:** 10.3389/fimmu.2025.1576016

**Published:** 2025-07-28

**Authors:** Zhiying Yin, Mengcheng Yin, Fei Zhao, Canya Fu, Wenjie Xu, Quanjun Fang, Xiaoying Gong, Guoping Cao, Canjie Zheng

**Affiliations:** ^1^ Department of Immunity, Quzhou Center for Disease Control and Prevention, Quzhou, Zhejiang, China; ^2^ Second Clinical Medical College, Tongji Medical College, Huazhong University of Science and Technology, Wuhan, Hubei, China; ^3^ Department of Epidemiology, School of Public Health, Zhejiang Chinese Medical University, Hangzhou, Zhejiang, China

**Keywords:** COVID-19, SARS-CoV-2, vaccination, infection, hybrid immunity

## Abstract

**Objectives:**

To investigate the factors influencing SARS-CoV-2 IgG antibody levels and protection in a population that has experienced both vaccination and COVID-19 infections, predominantly caused by the Omicron BA.5.2 and BF.7 variants.

**Methods:**

Anti-SARS-CoV-2 IgG antibody levels were measured using chemiluminescent microparticle immunoassay (CMIA). Multivariate regression analyses assessed factors influencing antibody levels, and decision tree models predicted variable priorities.

**Results:**

Among the 3494 participants, the median (IQR) anti-SARS-CoV-2 IgG level was 396.53 (280.51–471.03) AU/mL, with a seropositive rate of 97.28%. Multivariate analysis revealed that vaccination status, infection status, residence county, occupation, and vaccination interval were significantly associated with antibody concentration. The decision tree model indicated that vaccination had a greater effect on antibody concentration than infection, while infection had a stronger impact on seropositivity. The Breakthrough Infection group had the highest antibody concentration compared with other groups.

**Conclusions:**

Vaccination and infection were identified as the primary determinants of SARS-CoV-2 IgG antibody levels, with hybrid immunity significantly enhancing antibody responses. New evaluation methods or revised detection thresholds were needed to better assess population immunity.

## Introduction

Severe acute respiratory syndrome coronavirus 2 (SARS-CoV-2) has been spreading globally since December 2019. The virus has undergone numerous mutations, resulting in the emergence of various variants of concern (VOCs) from Alpha to Omicron, causing waves of the coronavirus disease 2019 (COVID-19) pandemic and posing a significant global health challenge (1). To minimize the hazards, the Chinese government’s COVID-19 prevention policy can be divided into four stages: “The stress response period”, “The COVID-19 prevention and control period”, “The normalized prevention and control period”, and “The overall adjustment period” (2). The main measures in the first three stages were the dynamic zero policy and COVID-19 vaccination, the former being a public health strategy that stopped community transmission as soon as SARS-CoV-2 was detected, and the latter creating an immune barrier against SARS-CoV-2 through multiple rounds of population-wide COVID-19 vaccination. At this stage, three types of COVID-19 vaccines were used in China: inactivated COVID-19 vaccine (vero cell), recombinant subunit COVID-19 vaccine (CHO cell), and adenovirus vector COVID-19 vaccine (Ad5). According to the National Health Commission of the People’s Republic of China, as of December 23, 2022, a total of 3.47 billion doses of COVID-19 vaccines have been reported, of which approximately 90% were inactivated COVID-19 vaccines (3). China, unlike many other countries with very high numbers of COVID-19 cases, had relatively few cases until December 2022 due to effective management (4). However, COVID-19 cases in China have grown rapidly since the fourth period with the adjustment of the dynamic zero COVID-19 strategy. Most COVID-19 cases presented with mild symptoms such as fatigue, cough, fever, and overall soreness, while others required hospitalization for more severe symptoms. Research has shown that higher antibody levels have reduced the severity of illness, such as high fever, diarrhea, chills, and anosmia (5).

The rapid evolution of the epidemic after the adjustment of the COVID-19 policy in China has been the focus of worldwide attention and concern that this outbreak may be driven by the emergence of new SARS-CoV-2 variants. Surveillance showed that both Omicron BA.5.2 and BF.7 were dominant in Beijing after November 14, 2022, accounting for 90% of local cases, with no evidence of novel variants (6). Among the sublines of Omicron, BA.5 and BF.7 have received particular attention due to their increased transmissibility, faster replication rate, and ability to evade immunity induced by prior infections or vaccinations. Infection with Omicron caused the lowest mortality compared to wild-type SARS-CoV-2 and the Alpha, Beta, and Delta variants (7). The co-circulation of BA.5.2 and BF.7 in Beijing could be seen as a snapshot of China, consistent with Quzhou surveillance. The population immunity could be derived from SARS-CoV-2 infection and/or COVID-19 vaccination, but little research has been conducted to identify the interactions.

Serum antibody testing has the advantages of uniform specimen collection, minimal laboratory requirements, and reliable results compared to nucleic acid testing. Both anti-spike (S) and anti-nucleocapsid (N) IgG antibodies were significantly elevated in subjects who never had a positive PCR result but were suspected of having SARS-CoV-2 infection, indicating that serologic testing can compensate for the shortcomings of PCR (8). Serum antibodies to SARS-CoV-2 induced by infection and/or vaccination have been associated with protection against severe disease for all VOCs so far, and serologic testing has been used to observe major shifts in the COVID-19 pandemic and resulting population immunity (9). Therefore, monitoring serum antibody levels in the population is crucial for assessing the impact of SARS-CoV-2 infections and/or vaccinations (10). In February 2023, we conducted a serologic survey among healthy individuals to investigate the factors influencing antibody responses, as well as the potential benefits of both infection and vaccination. This study may offer valuable insights into prioritizing hybrid immunity, thereby optimizing vaccination strategies.

## Methods

### Study design and participants

Quzhou is a prefecture-level city in Zhejiang Province in eastern China, which includes six counties and has a resident population of 2.28 million. Quzhou experienced the first pandemic wave of COVID-19 between December 2022 and January 2023, and more than 99% of the cases were first infections with the Omicron variant BA.5.2/BF.7 following the adjustment of the dynamic zero COVID-19 strategy in China. In February 2023, a cross-sectional study (Approval No. IRB-2023-R-001) was conducted in Quzhou using multi-stage stratified random sampling, firstly stratifying into counties and selecting local governments, then selecting urban or rural areas according to geographic location, next selecting the resident population in seven age groups (3-9, 10-17, 18-29, 30-39, 40-59, 60-79, and 80+ years). Participants were required to be over 3 years old, including non-household population residing in the local area for more than 6 months, excluding patients who were currently infected with SARS-CoV-2.

The sample size was estimated with the following formula: N=Z_α_^2^ *p*(1- *p*)/*d* ^2^, α=0.05, β=0.1, allowable tolerance *d*=12%, combined with the infection rate for sentinel surveillance of COVID-19 in Quzhou’s community-based population of January 26, 2023, *p*=82.56%, the eligible specimens of each age group in urban or rural areas was at least 40, and the cumulative sample size was 3,360 or more. A total of 3,543 individuals were recruited, and 46 of whom could not be queried for the history of COVID-19 vaccine, 3 of whom were less than 3 years old. Finally, 3,494 subjects were enrolled in the study.

### Investigation and blood sampling

Data collection consisted of face-to-face interviews and blood sample collection. A structured questionnaire including basic personal information, COVID-19 vaccination status (vaccination dose, time, and type), previous COVID-19 infections (whether or not infected, number of infections, time of latest infection, confirmed infection method, and clinical severity) was conducted by well-trained healthcare workers. Confirmed infection for COVID-19 included nucleic acid positivity, antigen positivity, or significant but undetected symptoms during the COVID-19 epidemic season. Approximately 3ml of blood sample was taken from each subject and immediately centrifuged at 3000 rpm for 5 minutes to separate serum, the serum was packed into 2ml screw-capped tubes and should not be left at room temperature for more than 4 hours, then transferred to store in a freezer below −80°C until analysis.

### Laboratory assays

The Quzhou Center for Disease Control and Prevention laboratory employed a chemiluminescent microparticle immunoassay (CMIA) to detect the presence of immunoglobulin G (IgG) antibodies against SARS-CoV-2 in serum specimens. The test procedure was carried out in strict accordance with the manufacturer’s guidelines, utilizing a two-step indirect immunoassay based on direct chemiluminescence technology. Step 1: Magnetic beads coated with a fusion antigen of SARS-CoV-2 nucleocapsid protein (N) and spike protein S1 (expressed using the baculovirus expression vector pFastBac1) were introduced to the sample. These coated beads formed antigen-antibody complexes with any IgG antibodies present in the serum specimen. Step 2: A curine anti-human IgG antibody acridine ester conjugate was added, which reacted with the pre-formed antigen-antibody complexes to create a stable antigen-antibody-secondary antibody complex. Step 3: Pre-excitation and excitation solutions were added to measure the relative luminous intensity (RLU). The level of IgG antibodies was directly proportional to the RLU value, which was then converted using a calibration coefficient determined by the calibrator.

The detection kit and iFlash 3000 analyzer utilized in this study were developed by Shenzhen Yahuilong Biotechnology CO., LTD. The kit had a detection range of 0 to 8000 AU/mL. The cut-off value was set at 10.06 AU/mL based on the highest point of Youden’s index, ensuring a specificity of 100% and a sensitivity of 98.5% for serum specimens. Specimens with results of ≥10 AU/mL were defined as seropositive for SARS-CoV-2 IgG antibodies, while those with results of <10 AU/mL were considered seronegative.

### Definition of vaccination status

According to the Technical Vaccination Recommendations for COVID-19 Vaccines in China, vaccination status was divided into the following five types (**Table 1**).

**Table 1:** The definition of vaccination status according to the technical vaccination recommendations for COVID-19 vaccines in China.

Vaccination status*	Definition
No Vaccination	No history of COVID-19 vaccination
Partial Vaccinated	Anyone of the following criteria: (1) One dose of Ad5 less than 14 days; (2) One dose of Vero or two doses of Vero less than 14 days from last dose; (3) One/two doses of CHO or three doses of CHO less than 14 days from last dose.
Primary Vaccinated	Anyone of the following criteria: (1) One dose of Ad5 or two doses of Vero or three doses of CHO more than 14 days from last dose; (2) Two doses of Ad5 less than 7 days from last dose; (3) Two doses of Vero + one dose of Vero/Ad5/CHO less than 7 days from last dose.
First Booster	Anyone of the following criteria: (1) Two dose of Ad5 more than 7 days from last dose; (2) Two doses of Vero + one dose of Vero/Ad5/CHO more than 7 days from last dose; (3) Three doses of Ad5 less than 7 days from last dose; (4) Two doses of Vero + one dose of Vero/Ad5/CHO + one dose of Vero/Ad5/CHO less than 7 days from last dose; (5) Three doses of CHO + one dose of Vero/Ad5/CHO less than 7 days from last dose.
Second Booster	Anyone of the following criteria: (1) Three doses of Ad5 more than 7 days from last dose; (2) Two doses of Vero + one dose of Vero/Ad5/CHO + one dose of Vero/Ad5/CHO more than 7 days from last dose; (3) Three doses of CHO + one dose of Vero/Ad5/CHO more than 7 days from last dose.

*Based on the immunization history of subject before the sampling date; Vero:inactivated COVID-19 vaccine (vero cell), CHO: recombinant subunit COVID-19 vaccine (CHO cell), Ad5: adenovirus vector COVID-19 vaccine (Ad5).

### Immunological source classification

Based on the COVID-19 infection and vaccination status of the participants, the source of immunity was categorized into four primary groups:

**Unvaccinated and uninfected**: This group comprises individuals who have neither received a COVID-19 vaccine nor been infected with the virus. They may possess a certain degree of innate immunity or have successfully avoided exposure to the virus altogether. Given their lack of exposure to the virus and absence of vaccination, their immunity to SARS-CoV-2 is either nonexistent or extremely limited.

Natural infection: This category includes those who have been infected with SARS-CoV-2 but have not received any COVID-19 vaccinations. Their immunity stems from their body’s natural response to the actual infection, providing them with a form of immunity that is specific to the virus.

COVID-19 vaccination: This group consists of individuals who have completed their COVID-19 vaccination series, with their last dose administered at least two weeks prior, and who have no documented history of SARS-CoV-2 infection. Their immunity is derived from the vaccine, which triggers an immune response without causing the disease, offering protection against the virus.

Breakthrough infection: These individuals have been vaccinated against SARS-CoV-2 but have subsequently contracted the virus. Although vaccines provide substantial protection, they are not infallible, and a small percentage of vaccinated individuals may still become infected. This group represents cases where the vaccine’s protective effect was not sufficient to prevent infection entirely.

### Statistical analysis

All data were double entered into Epidata 3.1 and exported to Microsoft Office Excel (version 2010) for analysis. The serum concentration of anti-SARS-CoV-2 IgG was presented as median and interquartile range (IQR), and statistical comparisons between groups were evaluated by Mann-Whitney U test or Kruskal-Wallis H test. The seropositive rates (SPRs) of different groups were examined by Chi-square test. Multiple linear regression and bivariate logistic regression were used to analyze antibody concentration and SPR, respectively. Decision Tree (DT) is a non-parametric technique designed to create a prediction model based on predictor variables, tailored to the characteristics of the target variable (11). In this study, we incorporated the CHAID technique into the DT to further predict, classify, and identify interactions between variables, using 70% random samples for modeling and the remaining 30% to test the model. Trend of antibody concentration from different immunity sources with age fitted by the locally weighted regression (Loess) method. Statistical analyses were performed with SPSS 27.0 (SPSS Inc., USA) and GraphPad Prism 10.2.3.403. Two-tailed *p <*0.05 was considered statistically significant.

## Result

### Demographic characteristics of study individuals

A total of 3494 eligible subjects were enrolled in this study. The total median (IQR) and SPR of anti-N/S1 IgG were 396.53 (280.51~471.03) AU/mL and 97.28% (3399/3494), respectively. For both antibody concentration and SPR, there were no differences by region (urban vs. rural) or gender (male vs. female), but the differences existed in other characteristics such as county, age group, occupation, education degree, vaccination status, infection frequency, infection interval, and vaccination interval (**Table 2**). There were also no differences in antibody concentration within different characteristics, for example, Changshan vs. Longyou (*p* =0.29) and Kaihua vs. Jiangshan (*p* =0.124) in county, 18–39 vs. 40-59 (*p* =0.357) and 40–59 vs. 60+ (*p* =0.346) in age groups, primary and lower vs. college and above (*p* =0.059) and secondary/technical school vs. college and above (*p* =0.113) in education degree, single vs. two+ times (*p* =0.601) in infection frequency, the interval of < 1 month, 1–3 months vs. ≥ 3 months after infection and uninfected vs. infected ≥ 3 months (*p* =0.971), unvaccinated vs. different intervals after vaccination and 3–6 months vs. 6–12 months after vaccination (*p* =0.542).

**Table 2:** The level of anti-SARS-CoV-2 IgG in people aged over 3 years in Quzhou, China.

Characteristics	No. of subjects	Median (IQR) (AU/mL)	*U/H*	*p*-Value	No. of positives	SPR (%)	*χ* ^2^	*p*-Value
County			715.29	<0.001			31.44	<0.001
Kecheng	638	414.76 (281.88~501.42)			620	97.18		
Qujiang	608	300.56 (196.55~397.38)			573	94.24		
Changshan	512	366.07 (188.58~426.72)			498	97.27		
Kaihua	559	476.94 (375.57~522.09)			546	97.67		
Longyou	616	373.49 (299.41~411.30)			606	98.38		
Jiangshan	561	483.63 (400.29~528.32)			556	99.11		
Region			1414560	0.07			0.15	0.70
Urban	1405	408.00 (268.93~478.84)			1365	97.15		
Rural	2089	390.41 (284.44~462.49)			2034	97.37		
Gender			1538515	0.67			<0.001	0.997
Male	1766	394.89 (279.78~468.89)			1718	97.28		
Female	1728	397.07 (280.87~472.11)			1681	97.28		
Age group (year)			23.68	<0.001			17.00	0.001
3-17	1003	416.12 (302.53~489.23)			966	96.31		
18-39	994	392.95 (298.17~460.66)			982	98.79		
40-59	524	384.10 (274.12~467.71)			514	98.09		
60+	973	381.72 (235.11~467.80)			937	96.30		
Occupation			46.06	<0.001			20.08	0.017
Student	1067	415.91 (310.12~488.86)			1028	96.34		
Farmer	883	376.40 (260.48~467.01)			860	97.40		
Worker	166	392.83 (288.22~452.24)			164	98.80		
Healthcare	183	420.34 (342.47~474.42)			182	99.45		
Teacher	25	399.83 (286.76~484.25)			25	100.00		
Officer	278	370.42 (256.37~437.54)			276	99.28		
Retirees	195	396.88 (266.01~481.22)			189	96.92		
Household	115	386.50 (280.64~449.29)			112	97.39		
Public service	155	407.43 (305.65~481.76)			147	94.84		
Others	427	380.64 (251.51~461.77)			416	97.42		
Education degree			14.66	<0.001			14.82	0.001
Primary and lower	1462	390.53 (238.34~470.97)			1404	96.03		
Secondary/ technical school	1136	402.94 (308.27~476.84)			1115	98.15		
College and above	896	395.40 (287.74~463.84)			880	98.21		
Vaccination status			360.53	<0.001			50.49	<0.001
No vaccination	138	43.70 (19.16~89.60)			119	86.23		
Partial vaccinated	64	121.06 (41.48~401.52)			60	93.75		
Primary vaccinated	1103	412.39 (309.33~482.32)			1067	96.74		
First booster	1625	380.45 (273.81~458.38)			1594	98.09		
Second booster	564	430.85 (370.36~503.57)			559	99.11		
Infection frequency			85.73	<0.001			81.66	<0.001
Uninfected	737	356.94 (81.84~448.96)			677	91.86		
One time	2737	403.78 (301.82~476.31)			2702	98.72		
Two+ times	20	416.02 (324.39~474.78)			20	100.00		
Interval from latest infection to sampling			86.59	<0.001			89.29	<0.001
Uninfected	739	357.30 (82.18~449.18)			679	91.88		
< 1 month	101	404.48 (251.47~490.78)			100	99.01		
1–3 months	2640	404.27 (302.56~476.04)			2607	98.75		
≥ 3 months	14	367.45 (229.91~415.42)			13	92.86		
Interval from latest vaccination to sampling			282.34	<0.001			45.11	<0.001
No vaccination	138	43.70 (19.16~89.60)			119	86.23		
< 3 months	649	422.06 (344.53~492.92)			641	98.77		
3–6 months	53	329.99 (123.83~479.93)			53	100.00		
6–12 months	614	374.45 (256.05~444.08)			593	96.58		
≥ 12 months	2040	403.47 (298.91~474.56)			1993	97.70		

### The multivariate analysis on anti-SARS-CoV-2 IgG by regression model

The results with no significant differences in the univariate analysis were merged, and further multivariate regression analysis was performed. The county of residence significantly influenced antibody levels, with Qujiang exhibiting the lowest antibody concentration and odds ratio (OR) relative to the reference group (Kaihua/Jiangshan). Age demonstrated a minor yet significant inverse correlation with both antibody concentration and OR, suggesting that older individuals tend to have marginally reduced antibody levels and odds. Education level did not markedly affect antibody concentration or OR. In contrast, occupation exerted a notable impact, with students presenting significantly elevated antibody concentrations but an insignificant OR. Public service workers, however, displayed a significant OR, indicating a decreased probability of high antibody levels. Vaccination status was a potent determinant of antibody levels, where unvaccinated individuals registered the lowest antibody concentration and OR. Infection status also emerged as a pivotal factor, with uninfected individuals showing significantly diminished antibody concentration and OR compared to those with a history of infection. The infection interval exhibited a minor and non-significant correlation with both antibody concentration and OR. Conversely, the vaccination interval showed a minor positive correlation with antibody concentration, but the OR was non-significant (**Table 3**).

**Table 3:** Multivariate analysis of anti-SARS-CoV-2 IgG using multiple linear regression and bivariate logistic regression models.

Characteristics	Antibody concentration (AU/mL)				SPR (%)		
	β*	95%CI for β		*p*-Value	OR*	95%CI for OR	*p*-Value
County				<0.001			<0.001
Qujiang	-144.3	-157.3~-131.3		<0.001	0.25	0.13~0.46	<0.001
Changshan/Longyou	-95.7	-106.5~-84.9		<0.001	0.82	0.43~1.57	0.54
Kecheng	-56.1	-68.8~-43.43		<0.001	0.43	0.21~0.86	0.02
Kaihua/Jiangshan	reference				reference		
**Age (year)**	-0.24	-0.58~0.10		0.162	0.98	0.96~1.00	0.02
Education degree				0.259			0.27
Primary and lower	4.60	-11.9~21.2		0.586	1.38	0.60~3.19	0.45
Secondary/technical school	10.6	-3.66~24.9		0.145	1.90	0.83~4.32	0.13
College and above	reference				reference		
Occupation				<0.001			0.13
Student	64.2	42.1~86.3		<0.001	0.69	0.23~2.12	0.52
Farmer	16.2	-1.07~33.4		0.066	1.98	0.81~4.83	0.13
Worker	10.8	-12.5~34.1		0.365	1.968	0.40~9.64	0.41
Healthcare	13.1	-11.1~37.4		0.290	2.138	0.24~18.8	0.50
Teacher	-39.7	-92.0~12.5		0.136	2.1*10^7^	0.00~	1.0
Officer	2.25	-18.0~22.5		0.827	3.11	0.63~15.3	0.16
Retirees	10.6	-13.9~35.1		0.396	1.69	0.52~5.50	0.38
Household	14.8	-12.0~41.7		0.278	1.37	0.33~5.63	0.66
Public service	5.75	-18.1~29.6		0.637	0.34	0.12~0.96	0.04
Others	reference				reference		
Vaccination status				<0.001			<0.001
No vaccination	-324.9	-351.1~-298.9		<0.001	0.07	0.02~0.20	<0.001
Partial vaccinated	-232.4	-269.3~-195.6		<0.001	0.23	0.04~1.20	0.08
Primary vaccinated	-107.6	-135.8~-79.4		<0.001	0.43	0.10~1.77	0.24
First booster	-73.0	-93.8~-52.3		<0.001	0.79	0.24~2.64	0.70
Second booster	reference				reference		
Infection status				<0.001			<0.001
Uninfected	-71.2	-89.9~-52.6		<0.001	0.13	0.06~0.29	<0.001
Infection	reference				reference		
Infection interval* **(month)**	-6.48	-16.2~3.29		0.194	0.87	0.58~1.30	0.49
Vaccination interval* **(month)**	1.66	0.11~3.21		0.035	0.98	0.91~1.04	0.39

*β: Regression coefficient; OR: Odds ratio. Infection interval: Interval from latest infection to sampling; Vaccination interval: Interval from latest vaccination to sampling.

### The interactions between characteristics using DT models

**Figure 1:**
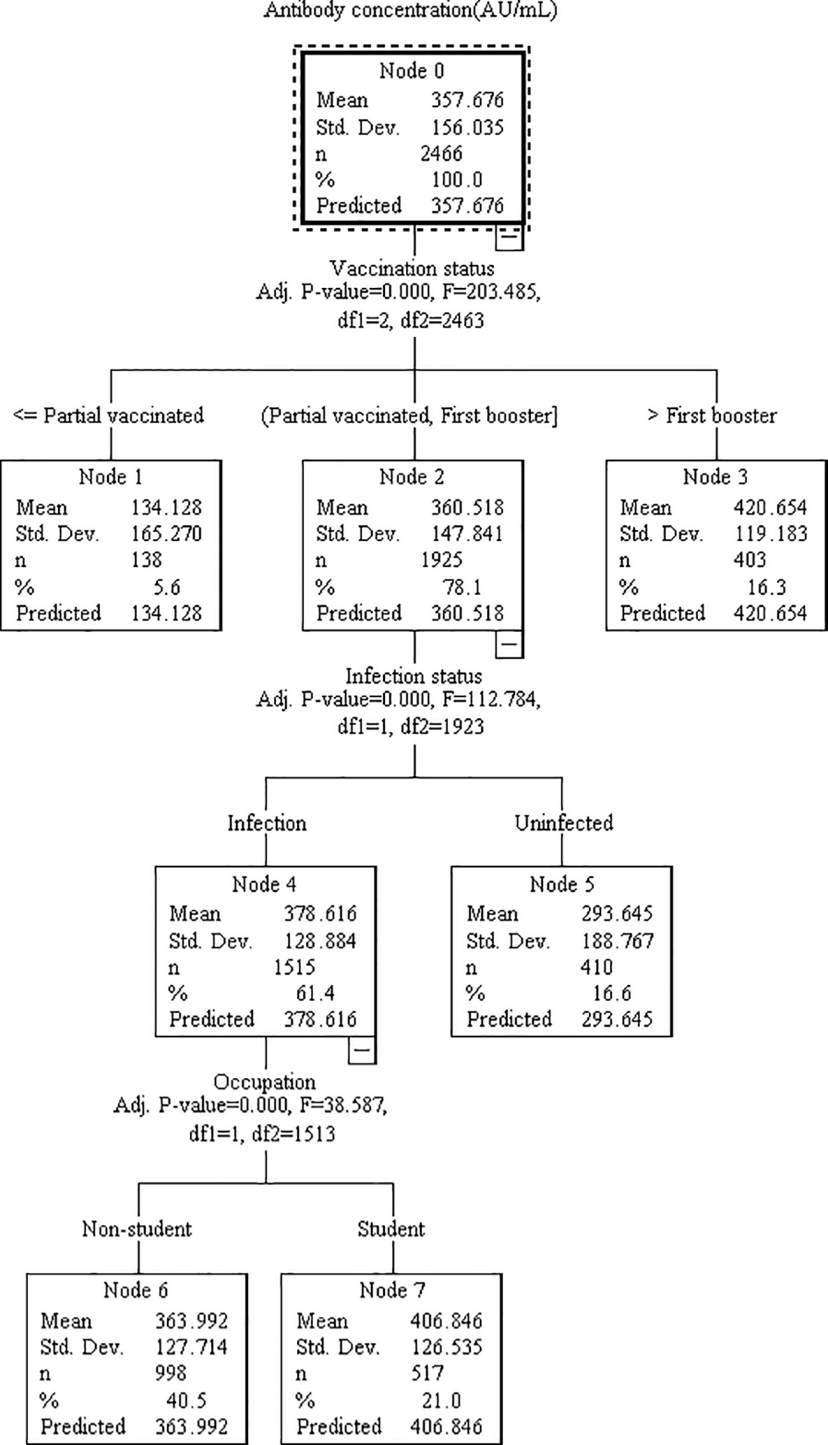
Decision tree model elucidating the relationship between antibody concentration and various associated features. The model was constructed utilizing the CHAID technique, employing a dataset of 3494 samples (with 70% randomly selected for model training and the remaining 30% reserved for validation). Each node signifies a decision point based on a specific feature, while the branches denote the corresponding outcomes. The features analyzed include vaccination status, infection status, and occupation.

**Figure 2:**
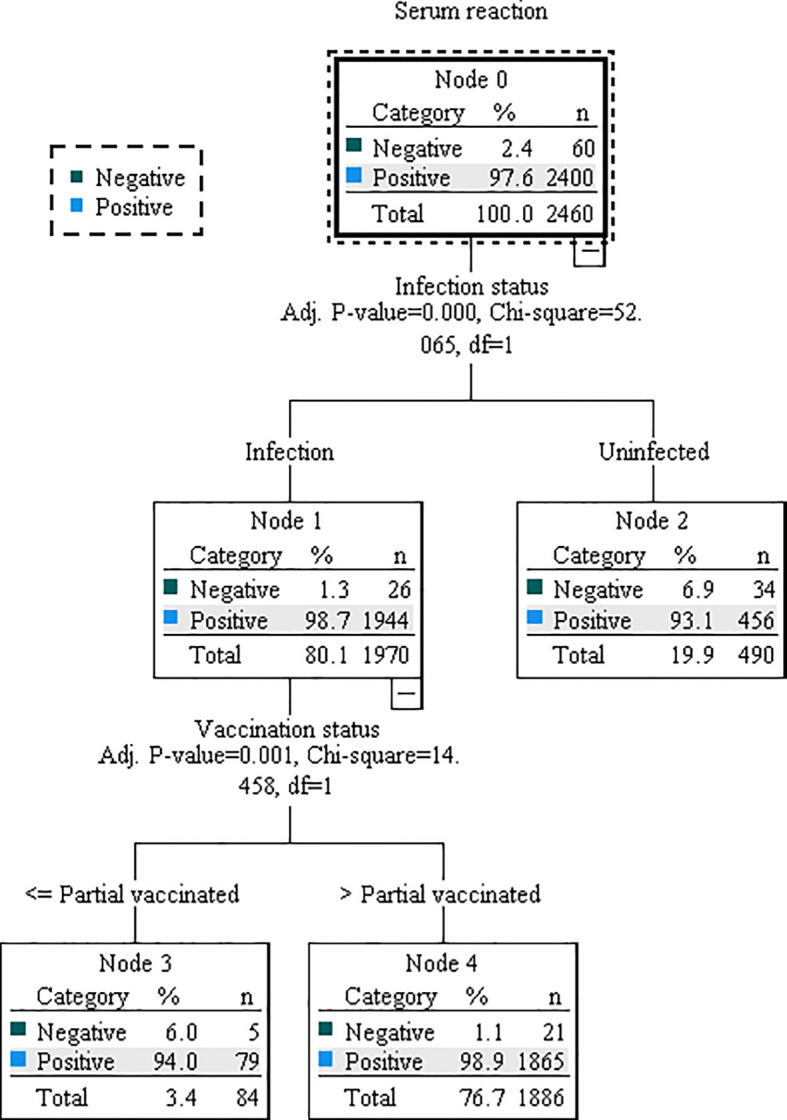
Decision tree model illustrating the association between serum reaction and relevant characteristics. The model was constructed using the CHAID technique, based on a dataset of 3494 samples. Of these, 70% were randomly selected for model training, while the remaining 30% were used for validation. Decision nodes and branches within the model are determined by key characteristics, including infection status and vaccination status.

Based on the results of multivariable regression analysis, we included the variables found to be associated as independent variables, and antibody concentration or serum reaction as dependent variable in the decision tree model for further analysis. Although both multiple linear regression and binary logistic regression showed that county of residence affected antibody levels, this may be due more to selection bias and was not included in the model. The DT model for antibody concentration was divided into 3 layers with 5 terminal nodes. Vaccination status, infection status, and occupation were the influencing factors, among which vaccination status had the greatest influence, followed by infection status (**Figure 1**). Those who received primary vaccination or first booster, infection and non-student accounted for the largest (40.3%) with a predicted antibody concentration of 363.92 AU/mL. The highest predicted antibody concentration was 420.65 AU/mL for second boosters, representing 15.7%. The DT model for serum reaction was divided into 2 layers with 3 terminal nodes, infection and vaccination status were the impacting factors, and infection had a greater effect than vaccination (**Figure 2**). The probability of being positive was highest in those infected and completed primary vaccination or more, accounting for 75.7%.

### Anti-SARS-CoV-2 IgG from different sources

**Figure 3:**
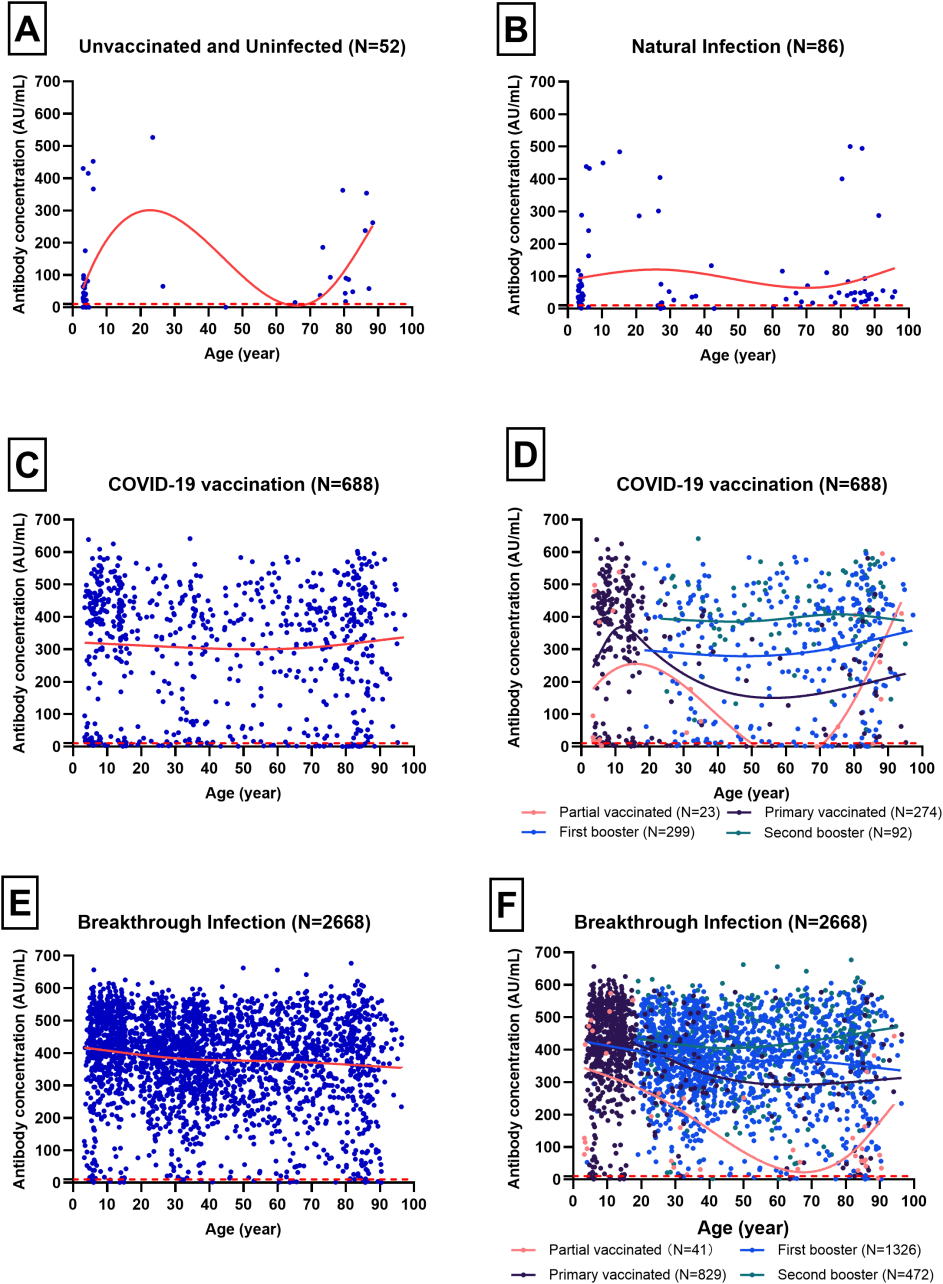
Trends of antibody concentration with age for different immunity sources, analyzed using the Loess smoothing method. Blue scatter points represent individual samples, while red curves depict the trend of antibody concentration with age. Different colored curves correspond to various COVID-19 vaccination statuses, and the red dotted line indicates the cut-off value of 10 AU/mL. **(A)** Trend of antibody concentration for Unvaccinated and Uninfected with age(N=52). **(B)** Trend of antibody concentration for Natural Infection with age(N=86). **(C)** Trend of antibody concentration for COVID-19 Vaccination with age(N=688). **(D)** Trend of antibody concentration with age in different vaccination status of COVID-19 Vaccination(N=688). **(E)** Trend of antibody concentration for Breakthrough Infection with age(N=2688). **(F)** Trend of antibody concentration with age in different vaccination status of Breakthrough Infection(N=2688).

The median (IQR) antibody concentrations for the Unvaccinated and Uninfected, Natural Infection, COVID-19 Vaccination, and Breakthrough Infection groups were 42.67 (16.20~92.20) AU/mL, 44.97 (21.29~84.99) AU/mL, 373.49 (159.07~519.69) AU/mL, 407.50 (315.31~478.14) AU/mL, respectively (**Figure 3**). Apart from no significant difference between the Unvaccinated and Uninfected and Natural Infection groups, the other groups exhibited an upward trend in antibody concentrations. **Figures 3A, B** revealed that the age distribution of the Unvaccinated and Uninfected, as well as the Natural Infection populations, is predominantly concentrated in individuals under 10 years old and those over 60 years old. In the COVID-19 Vaccination population, the median (IQR) antibody concentrations for individuals with partial vaccination, primary vaccination, first booster, and second booster were 95.03 (17.00~410.03) AU/mL, 374.17 (69.90~451.47) AU/mL, 349.71 (157.79~443.85) AU/mL, 414.04 (340.04~494.52) AU/mL, respectively. Notably, the antibody concentration in the second booster group was significantly higher than that in the other vaccination status groups, with no significant differences observed among the other vaccination status groups (**Figures 3C, D**). In the Breakthrough Infection population, the median (IQR) antibody concentrations for individuals with partial vaccination, primary vaccination, first booster, and second booster were 127.35 (56.39~385.38) AU/mL, 421.53 (343.07~496.11) AU/mL, 384.45 (290.14~461.95) AU/mL, 432.61 (374.30~503.95) AU/mL, respectively (**Figures 3E, F**). Statistically significant differences in antibody concentrations were found among the four vaccination status groups (*p <*0.05).

## Discussion

Previous studies have shown that anti-S and anti-N IgG antibodies are strongly associated with the risk of SARS-CoV-2 infection and can serve as markers for infection protection and symptom severity (12, 13). The conventional neutralization test, using live viral particles, is the gold standard for measuring neutralizing activity in sera. However, it has major drawbacks: it is time-consuming, requires a biosafety level 3 laboratory (limiting accessibility), and is difficult to standardize and scale up for widespread use. Moreover, a recent study involving individuals with a history of COVID-19 vaccination and/or SARS-CoV-2 infection demonstrated that IgG titers measured by chemiluminescent microparticle immunoassay (CMIA) can provide valuable insights into the immune response (14). CMIA-measured IgG levels may offer predictive value for infection severity and immune protection, particularly in the context of hybrid immunity. Future research should directly link CMIA-measured IgG titers to neutralizing antibody levels and clinical outcomes, clarifying their significance against emerging SARS-CoV-2 variants.

The overall median IgG concentration was found to be 396.53 AU/mL with an IQR of 280.51-471.03 AU/mL, and the SPR was 97.28% (3399/3494). The high SPR of our study indicated that the majority of the population studied had been exposed to the SARS-CoV-2 virus or had received a vaccination, leading to the development of detectable IgG antibodies (15). Univariate analysis revealed no significant differences in either the median IgG concentration or the SPR when comparing urban versus rural regions or male versus female subjects, consistent with some research (16). Multivariate analysis further excluded the effect of education level on antibody concentration and SPR, indicating that socioeconomic factors might not play a substantial role in this context (17). The residence county showed significant effects, with Qujiang exhibiting the lowest antibody concentration and odds ratio (OR) relative to the reference group (Kaihua/Jiangshan). The timing and coverage of vaccination campaigns can affect the overall immune status of the population. Among the participants, the proportion of Qujiang who did not receive the second booster in time was larger, while the proportion of Changshan who had no history of immunization was larger.

Interestingly, age demonstrated a minor yet significant inverse correlation with both antibody concentration and OR, suggesting that older individuals might have marginally reduced antibody levels and odds. This could be due to age-related immune decline (18, 19). The scatter plots revealed interesting age-related trends in antibody concentrations. For instance, the Unvaccinated and Uninfected group and Natural Infection group showed a bimodal distribution with peaks in younger and older age groups. This could reflect higher susceptibility and exposure risks in these demographics. In contrast, the COVID-19 Vaccination and Breakthrough Infection groups exhibited a more uniform distribution across age groups, indicating that vaccination helps in achieving a broader immune coverage regardless of age (20). Additionally, occupation played a notable role, with students showing significantly elevated antibody concentrations, although the OR was not significant. In contrast, public service workers displayed a significant OR, indicating a decreased probability of high antibody levels, which might be influenced by occupational exposure risks and preventive measures (21). Healthcare workers, public service workers, and students may have different levels of exposure to the virus depending on their job responsibilities and the settings in which they work (22).Our analysis revealed that antibody concentrations vary significantly across different groups. Notably, the COVID-19 Vaccination and Breakthrough Infection groups exhibited higher median antibody concentrations compared to the Unvaccinated and Uninfected and Natural Infection groups. This suggested that both vaccination and exposure to the virus can effectively boost immune responses, as reflected by higher antibody levels (23). Infection triggers mainly S2/N-terminal domain (NTD) reactive antibodies, whereas vaccination induces primarily anti-receptor binding domain (RBD) antibodies, their combination is called hybrid immunity (24). Previous research has shown that hybrid immunity was potentially mediated by T-cell induced immunity, which was less affected by mutations in the VOCs that have evolved to date (25). The decision tree model highlighted the critical role of vaccination status in determining antibody concentrations. Individuals who received a second booster showed the highest predicted antibody concentration, significantly higher than those with partial vaccination or first booster. This finding emphasized the importance of booster doses in enhancing immunity against SARS-CoV-2 (26). Infection status emerged as another pivotal factor affecting antibody levels. Compared to those who have not been infected, Individuals who have been infected have significantly higher antibody levels. This could be attributed to the natural immune response triggered by the infection, which complements the response induced by vaccination (27). However, the frequency of infection and the time since infection exceeding 3 months have no impact on antibody concentrations. This suggested that the immune response to natural infection may wane over time, potentially leaving individuals susceptible to reinfection (28). Hybrid immunity produced stronger adaptive immunity than those vaccinated or infected alone (29, 30).

Neither vaccinations nor infections seemed to thwart SARS-CoV-2 for long (31). Although anti-SARS-CoV-2 IgG antibody was regarded as a marker of infection protection (32), our study showed a limited role. In our study, breakthrough infections constituted 76.36% of the cases, with the SPR of 97.28%. A threshold of 10 AU/mL based on Youden’s index to define seropositivity in this study. The Youden’s index, calculated as J=sensitivity+specificity−1, identifies the point on the Receiver Operating Characteristic (ROC) curve, which are a common method for determining optimal cut-off points in diagnostic tests. However, this threshold may be limited by the specific population and conditions under which it was derived. The Omicron variant has been shown to elicit different antibody responses compared to earlier variants, which may affect the sensitivity and specificity of the chosen threshold (33). Antibody assays can be used for estimating SARS-CoV-2 infection prevalence in areas where spike-based COVID-19 vaccines was used, but poor sensitivity in detecting infection after vaccination has been reported (34). ROC analysis results suggested that the cutoffs in Japan may be lower than the manufacturer’s cutoff (10 AU/mL) (35). Therefore, the validation of this threshold in populations with high prevalence of Omicron infections is crucial to ensure its accuracy and reliability. While the use of Youden’s index to derive a 10 AU/mL threshold for defining seropositivity is a well-established method, its application in the context of variant-specific immune responses and hybrid immunity requires further validation. Alternative cut-offs should be considered to improve the accuracy of seroprevalence interpretations, especially in populations with diverse immune profiles.

While this study provides valuable insights, it has some limitations. First, the models in our study do not fully capture the kinetics of antibody waning. The cross-sectional data only obtained a single serum antibody data from individual, and the stringent prevention and control measures meant that the vaccination and infection timelines were essentially synchronized, with no significant intervals. Second, the study did not account for variant-specific immune responses, which could influence antibody levels and protection against different strains of SARS-CoV-2. Finally, future research should also explore the impact of hybrid immunity on antibody levels and protection. Moreover, understanding the correlates of protection, such as T-cell responses and neutralizing antibodies, will be crucial for developing effective vaccination strategies and public health policies.

In conclusion, this study demonstrated that vaccination and breakthrough infections significantly enhanced antibody levels against SARS-CoV-2. The findings emphasized the importance of booster doses and highlighted the influence of infection status and occupation on immune responses. Understanding these factors can inform vaccination strategies and public health interventions to optimize immunity and control the spread of COVID-19. Further research was needed to explore new evaluation methods or to modify the existing detection threshold.

## Data Availability

The raw data supporting the conclusions of this article will be made available by the authors, without undue reservation.
